# Digital inclusion as a social determinant of health

**DOI:** 10.1038/s41746-021-00413-8

**Published:** 2021-03-17

**Authors:** Cynthia J. Sieck, Amy Sheon, Jessica S. Ancker, Jill Castek, Bill Callahan, Angela Siefer

**Affiliations:** 1 Department of Family Medicine, The Ohio State University College of Medicine, Columbus, OH USA; 2 The Ohio State University Center for the Advancement of Team Science, Analytics, and Systems Thinking, Columbus, OH USA; 3 Case Western Reserve University School of Medicine, Clinical and Translational Science Collaborative, Cleveland, OH USA; 4 Weill Cornell Medical College, Department of Healthcare Policy & Research, Division of Health Informatics, New York City, NY USA; 5 College of Education, Department of Teaching, Learning, and Sociocultural Studies, University of Arizona, Tucson, AZ USA; 6 National Digital Inclusion Alliance, Columbus, OH USA

**Keywords:** Health services, Interdisciplinary studies

The use of digital tools and applications is steadily increasing and can support a range of health information needs^1–3^. As tools such as patient portals, health trackers, and remote monitoring devices see greater use, research suggests that tools such as health apps and patient portals can foster greater patient engagement, better support for patients outside of the clinic visit, and can improve health outcomes^3–9^. However, greater reliance on digital tools has the potential to increase disparities between those who have skills and access to digital tools and those who do not and thereby existing health disparities.

According to a recent Brookings Institution report, 15–24% of Americans lack any sort of broadband connection to the Internet with which to use mobile health technology. These differences only increase when examining the issue by income groups: 38% of households earning less than $20,000 lack a broadband subscription^10^. The digital divide by income exists in both rural and urban areas. As practitioners working at the intersection of digital inclusion and health, we would like to highlight some less visible dimensions of the digital divide and offer suggestions to facilitate digital inclusion and ensure equitable and impactful adoption of mobile health technologies.

**Fig. 1:**
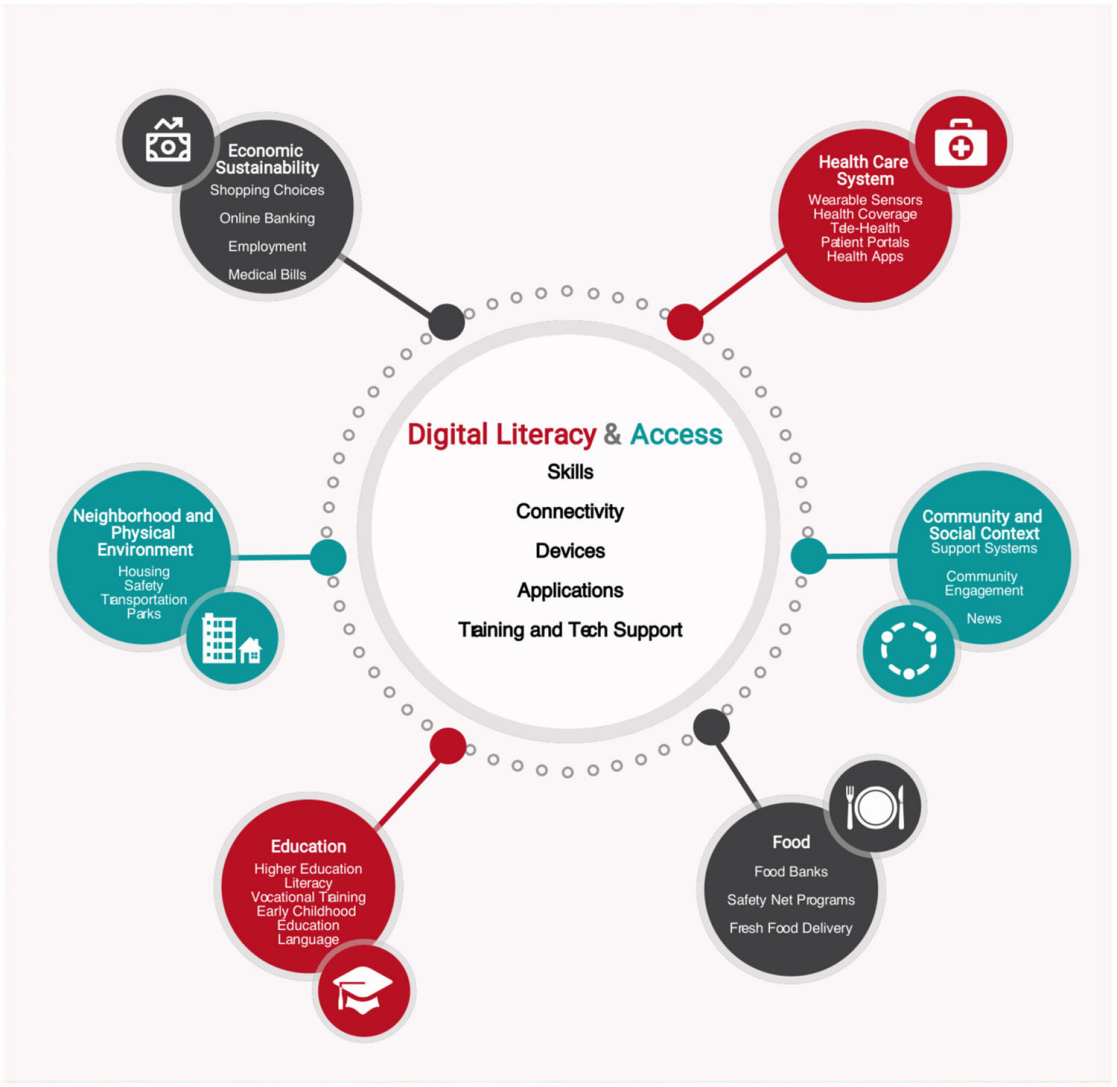
Digital literacies and social determinants of health. Digital literacy and access, including skills, connectivity, devices and training and technical support, relate to all other domains of social determinants of health.

Digital literacies and Internet connectivity have been called the “super social determinants of health” because they address all other social determinants of health (SDOH), as shown in Fig. 1^11^. For example, applications for employment, housing, and other assistance programs, each of which influences an individual’s health, are increasingly, and sometimes exclusively, accessible online. The costs of equipping a person to use the Internet are substantially lower than treating health conditions and the benefits are persistent and significant^12^, making the efforts to improve digital literacy skills and access valuable tools to reduce disparities.

With these challenges in mind, we offer the following recommendations. First, healthcare systems should adopt a digital inclusion-informed strategy regarding mobile health that (1) recognizes their community’s level of access to devices and Internet connectivity and (2) supports patients in their initial and sustained technology use. Digital inclusion refers to the activities necessary to ensure equitable access to and use of information and communication technologies, including (1) affordable broadband Internet service, (2) Internet-enabled devices, (3) access to digital literacy training, (4) quality technical support, and (5) applications and online content designed to enable and encourage self-sufficiency, participation, and collaboration^13^. These form the foundation for use of mobile technology in healthcare. While knowing whether an individual’s access is important, it is vital for health systems to understand the larger environment shaping patients’ digital experience. Adoption rates are nearing ubiquity among highly educated individuals with at least moderate income, but important pockets of nonadoption remain.

Most mobile health technology requires a data plan and/or home broadband, yet the American Community Survey shows that 40% of low-income households lack a subscription, requiring them to use limited cell plan data or local public wifi hotspots^12^. These options may appear affordable but they contain important limitations. Using prepaid plans, patients may run out of data or need to prioritize data for specific uses. Even with their lower cost, they may still be unaffordable, particularly for families in need of multiple devices. Open wifi access points are another option but may only be available in public locations in which patients may feel uncomfortable accessing their personal health information.

Prior to the rapid increase in telehealth use due to COVID-19, patient portals to their electronic health record (EHR) were the most common form of mobile health and a gateway to other mobile health applications. However, studies show that lack of Internet access is a leading factor inhibiting use of patient portals^14^. Smartphones may seem to be a logical and ubiquitous substitute for home Internet, but significant gaps still exist for rural, poor, and older adults. Research shows that nearly one-half of older adults and 30% of those earning less than $30,000 own a smartphone and many low-income households share devices, raising both access and privacy issues^15^. Understanding the nuances of access in the communities they serve can help healthcare systems implement more inclusive strategies.

Digitally inclusive strategies of health system adoption also support patients in their use of technology at all levels and should include digital skill training, particularly for recent adopters of technology or those who may have devices with limited features. Patients may also need assistance with setting up email and patient portal accounts. In addition, it is critical to provide ongoing support for patients, reduce medical jargon, and provide interpretive resources, and ensure that technology and training are offered equitably to all patients, not just to those who are confident enough to request help^16^.

Second, we recommend systematically assessing individual patients’ access and digital literacies. This became particularly clear since the rapid and pervasive shift to telehealth during the COVID-19 pandemic. Simply asking patients what devices they own and how they access the Internet is not typical in the clinical context, but this information can shape the type of technology a clinician can recommend. The lack of routine assessment prior to COVID-19 meant that some patients fell between the cracks as care shifted to nearly all virtual^17^. Incorporating this and other SDOH into the EHR encourages more consistent documentation and allows assessment of population-level metrics of access^18^. When digital skill and connectivity gaps are assessed systematically and universally, a health system can document overall population-level metrics, examine disparities, and track changes over time.

Third, health systems should partner with community organizations with expertise in training in digital literacy skills and facilitating connectivity. Libraries not only offer the Internet but also provide a spectrum of training services from basic digital literacies to skills required for specific devices and applications. Some communities have leveraged community health workers and patient navigators to screen and refer patients for gaps in basic digital literacies and connectivity^19,20^. They can provide hands-on training in the use of mobile health technologies for patients who do have adequate digital access. Allied health professional education programs leverage a “train the trainer” model to prepare the future healthcare workforce to undertake these tasks^21–23^. The National Digital Inclusion Alliance (NDIA) offers a comprehensive list of organizations across the country that provide digital literacy training and national and local resources for free/low-cost Internet and computers^13^.

Mobile health technologies hold significant promise to increase the efficiency of care and improve health outcomes. Yet, we must be cognizant of their potential to increase health disparities. National efforts have been undertaken to promote broadband, such as the Federal Communications Commission’s (FCC) Lifeline program that subsidizes the cost of smartphones and Internet service for low-income individuals^24,25^. However, the Lifeline Program’s impact is limited by low consumer awareness, and the qualification process varies by state and by the service provider. In addition, Internet service may still be unaffordable even with the monthly subsidy. Another program, the Federal Broadband Opportunities Program, supported over 4 million people to get online for the first time with a $4 billion program but those one-time dollars are long gone, leaving a gap in the need for adult digital literacy support. BTOP has only two remaining operational programs with no new funding on the horizon^25,26^. In response to the current COVID-19 pandemic, the FCC also introduced a variety of programs to increase Internet access for the use of telehealth, including paying for devices and access. However, the future of these programs after the COVID-19 pandemic is unclear^13^. As clinical care incorporates more technology in more contexts, we suggest the recommendations above to facilitate equitable adoption of mobile health technology.
